# Extracellular vesicles and oncogenic signaling

**DOI:** 10.1002/1878-0261.12855

**Published:** 2020-12-06

**Authors:** Antonia Schubert, Michael Boutros

**Affiliations:** ^1^ Division Signaling and Functional Genomics German Cancer Research Center (DKFZ) and Heidelberg University Germany; ^2^ Clinic for Hematology and Medical Oncology University Medical Center Göttingen Germany

**Keywords:** exosomes, extracellular vesicles, metastasis, microvesicles, signaling, tumor progression

## Abstract

In recent years, extracellular vesicles (EVs) emerged as potential diagnostic and prognostic markers for cancer therapy. While the field of EV research is rapidly developing and their application as vehicles for therapeutic cargo is being tested, little is still known about the exact mechanisms of signaling specificity and cargo transfer by EVs, especially *in vivo*. Several signaling cascades have been found to use EVs for signaling in the tumor–stroma interaction. These include potentially oncogenic, verbatim transforming, signaling cascades such as Wnt and TGF‐β signaling, and other signaling cascades that have been tightly associated with tumor progression and metastasis, such as PD‐L1 and VEGF signaling. Multiple mechanisms of how these signaling cascades and EVs interplay to mediate these complex processes have been described, such as direct signal activation through pathway components on or in EVs or indirectly by influencing vesicle biogenesis, cargo sorting, or uptake dynamics. In this review, we summarize the current knowledge of EVs, their biogenesis, and our understanding of EV interactions with recipient cells with a focus on selected oncogenic and cancer‐associated signaling pathways. After an in‐depth look at how EVs mediate and influence signaling, we discuss potentially translatable EV functions and existing knowledge gaps.

## Graphical abstract

Extracellular vesicles (EVs) emerged as potential diagnostic and prognostic markers for cancer therapy. This review summarizes mechanisms of signaling specificity and cargo transfer by EVs in the oncogenic and cancer‐associated signaling cascades Wnt, TGF‐β, ErbB, VEGF, and PD1–PD‐L1. Translatable EV functions and existing knowledge gaps are discussed to possibly further exploit EVs for diagnostics and therapeutic approaches in the future.

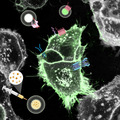

**Abbreviations**
EGF(R): epidermal growth factor (receptor)
EMT: epithelial–mesenchymal transition
EP(s): extracellular particle(s)
EV(s): extracellular vesicle(s)
Exo(s): exosome(s)
Her: human epidermal growth factor receptor
ISEV: International Society for Extracellular Vesicles
MSC: mesenchymal stem cells
MV(s): microvesicle(s)
MVB: multivesicular body
PD‐L: programmed death‐ligand
TGF‐β: transforming growth factor‐β
VEGF: vascular endothelial growth factor
Wnt: wingless and Int/wingless‐related integration site

## 1 Introduction

Extracellular vesicles (EVs) are small, lipid bilayer components that are released from many cell types []. They can transport signaling‐active molecules in the form of proteins, DNA/RNA, lipids, and metabolites between cells of different origins [1]. In the last years, research on EVs has received increased attention because of their wide‐ranging biological properties and their abilities to govern physiological and pathophysiological processes such as immune responses, tissue regulation, and organ remodeling [3]. Furthermore, EVs have been proposed as tools for diagnostics and for the therapy of various diseases such as cardiovascular and neurodegenerative diseases, and cancer [4, 5].

Recent reviews have highlighted the role of EVs in cancer including on EV biogenesis and function [3], their role in tumor biology, tumor progression, and metastasis [6, 7], EVs in the tumor microenvironment [8, 9, 10, 11, 12], EVs as biomarkers [13], and their artificial production for therapeutic applications [5, 14, 15]. In this review, we focus on the various mechanisms of EV‐mediated signaling in the context of cancer, while pointing out common features, gaps in the current knowledge, and potentially translatable applications of EVs in cancer treatment.

## 2 Extracellular vesicles

### 2.1 Definition and biochemical properties of EVs

Extracellular vesicles are defined as lipid bilayer‐surrounded particles, naturally released from cells [2]. As EVs are released from both pro‐ and eukaryotic cells, they represent a conserved and universal biological structure with the capability to pass material and information across biological barriers [16]. With increasing numbers of studies on EVs in the past years, the multitude of overlapping and partly unclear definitions of EV subpopulations and their nomenclature are an ongoing concern [2]. Due to mostly impure isolation techniques and a lack of standards, it has remained difficult to distinguish distinct EV populations with regard to their varying intracellular origin and to the mechanisms of cargo sorting and EV secretion [2, 3, 17, 18].

To reach a consensus about EV definitions, a nomenclature has been developed in recent years to describe and better compare experimental results [2, 18]. The International Society for Extracellular Vesicles (ISEV) suggests an in‐depth characterization of the respective EV fractions by defining: their (a) physical characteristics (e.g., sizes or densities with defined ranges); (b) biochemical composition of the isolated fractions (e.g., by markers such as CD63/81^+^); and (c) in‐depth description of how EVs were isolated (e.g., cells of origin, biological fluids) [2].

The smallest size of membranous vesicles is predicted to be 10–20 nm, which is determined by the membrane’s thickness and its phospholipid composition [19]. Independent of their subcellular origin, one can distinguish between small (sEV) and large (lEV) EVs, ranging up to and over 100–200 nm, respectively [2]. In case of unclear characteristics, it was suggested to use alternative terms such as ‘extracellular particle’ (EP) [2].

**Fig. 1:**
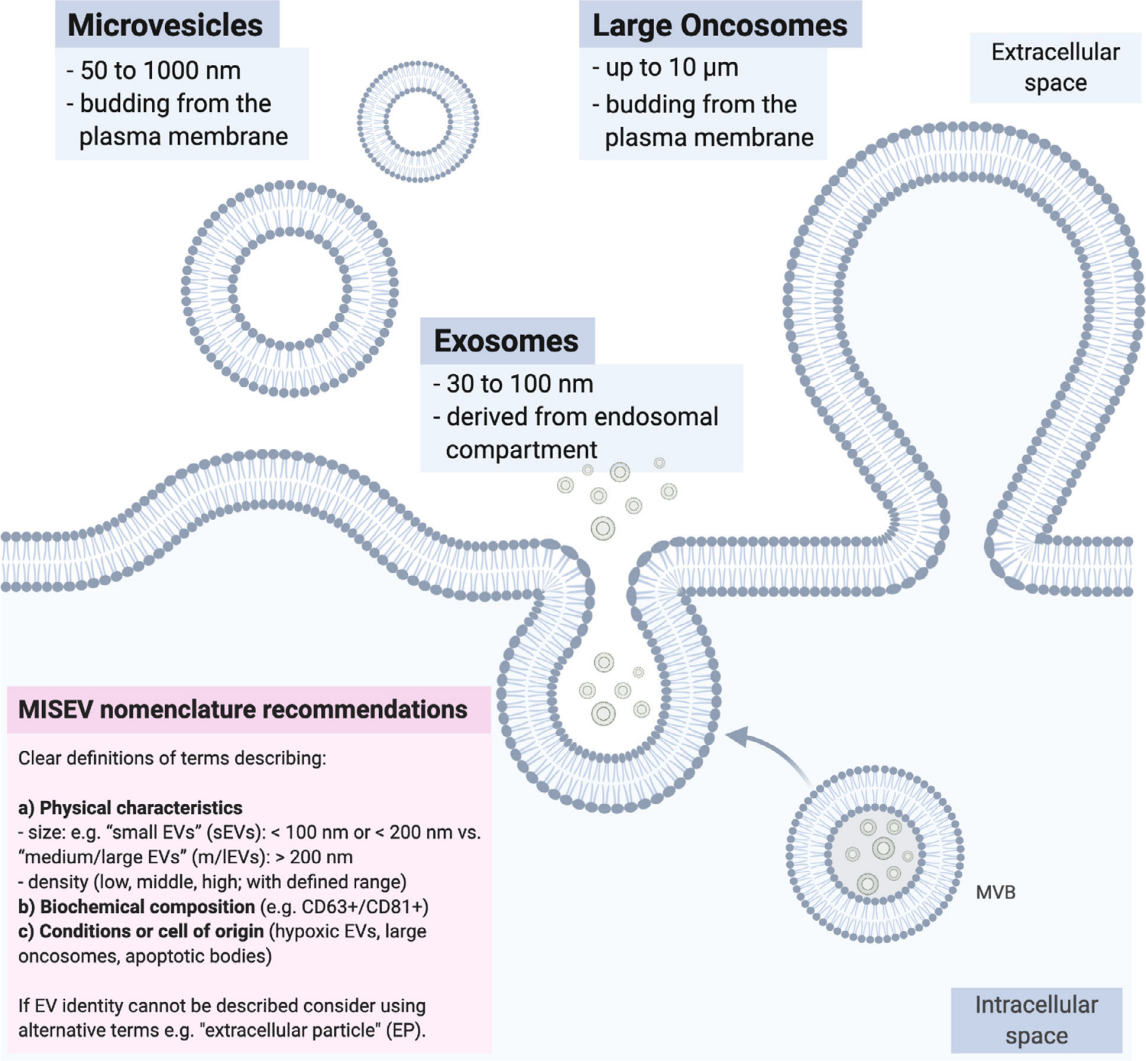
EV nomenclature. Size and cellular origin of different EV subpopulations with regard to commonly used terminology are schematized. Since EVs are heterogeneous and current isolation techniques depend on EV size, density, or insufficiently characterized surface markers, EV fractions obtained during the different isolation procedures are most likely impure mixtures of various EV fractions, for example, Exos and MVs. Therefore, current recommendations of the ISEV in the MISEV consensus are aiming to consolidate common classification standards for EV research in the future (see pink box as published in [2]). MISEV, Minimum Information for the Study of Extracellular Vesicles.

While definitions remained fluid, several terms were often used to describe EVs (Fig. 1): Exosomes (Exos; 30–100 nm) are an EV population that originates from multivesicular bodies (MVBs) in the endosomal compartment [1, 3]. Cargo sorting onto and into Exos can take place either by endosomal sorting complex required for transport (ESCRT)‐dependent or ESCRT‐independent cargo clustering. EV cargo is sorted onto MVB membranes, and intraluminal vesicles form by invagination of the MVB membrane [1, 3]. Through fusion of endosomal membranes with the outer plasma membrane, Exos are released into the extracellular space [1, 3]. Microvesicles (MV or ectosomes/microparticles) with a size ranging from 100 to 1000 nm are formed by direct budding from the outer plasma membrane [3, 20]. Based on this definition, oncosomes (transferring oncogenic cargo [21]), large oncosomes (an especially large subset of cancer cell‐derived EVs shed by migrating prostate cancer cells [22, 23]), and apoptotic bodies (from dying cells), which can be up to multiple micrometers in size, can be classified as specific EV subtypes [24]. Less characterized are EVs originating from other migrating cells, which can be called migrasomes [25].

In 2018, Zhang *et al*. [26] described a new EP fraction by employing asymmetric flow field‐flow fractionation (AF4) for EV isolation. These ~ 35 nm nonmembranous and therefore by definition non‐EV nanoparticles were termed exomeres [26]. The identification of exomeres demonstrates how the advancements in isolation technologies or adaptations of preexisting protocols contribute to the identification of new EV subpopulations with potentially new and distinct biological functions [27]. Structured comparisons of EVs not only with exomeres but also other non‐EV nanoparticles, such as high‐density lipoproteins and low/very‐low‐density lipoproteins are a prerequisite to fully understand signaling via EVs in the future [28].

Although the exact mechanisms of EV–cell interaction and cargo transfer are not fully understood, multiple processes have been implicated in mediating the EV–cell interaction and cargo transfer: (a) surface binding and potentially direct signaling induction; (b) EV internalization; and (c) membrane fusion [1, 3].

Extracellular vesicles–cell interactions are mediated by integrins and other surface markers such as tetraspanins, lipids, lectins, and heparan sulfate proteoglycans [3]. Direct EV–cell interaction was described via integrins or tetraspanins with intracellular adhesion molecules [29]. Additionally, lipid‐mediated direct cell targeting by recruitment of lipid‐binding proteins (such as galectin‐5) through phosphatidylserine was observed [30]. However, EVs can also interact indirectly with recipient cells through linking components of the extracellular matrix (ECM) [31]. ECM components such as fibronectin have also been described as important for EV‐controlled directional cell movements, especially migratory speed [32].

Bagi *et al*. [33] further reported a coupling role of microparticle‐ and EV‐carried arginine–glycine–aspartate proteins and peptides by binding to the vascular endothelium mediating consecutive platelet adhesion. This suggests a link of EV function in the endothelium in pathological processes such as stroke, cerebral inflammation, and cerebral tumors [33]. Additionally, signaling by EV‐transported proteins can also take place without EV uptake or content delivery, as is the case with direct activation of T cells by MHC Class II molecule‐presenting EV [34].

EV internalization can occur through unspecific processes such as macro‐ or micropinocytosis depending on the physiological properties of EVs and the recipient cells [35, 36]. In addition, clathrin‐dependent endocytosis and clathrin‐independent endocytosis have been implicated in EV uptake [37, 38]. Most likely, endosomal escape will be a prerequisite at least for some of the signaling‐active cargo such as RNA [39]. Nevertheless, signaling activation from within the endosomal compartment after Exo uptake has recently been reported by Shelke *et al*. [40]. EV cargo can be released into the cytoplasm, targeted to the lysosome for degradation, or recycled back to the plasma membrane for discharge back into the extracellular compartment with potentially changed cargo loading [41].

As a further route of cargo delivery, direct membrane fusion between EVs and the outer cell membranes has been proposed, even though most studies describe internalization of EV as the main mechanism of cargo delivery to the endosomal compartment [1, 42].

When studying EV–cell interactions, a well‐described effect from nanomedical studies is important to consider: In biological fluids such as blood, the interaction between nanoparticles and macromolecules at the nanoplasma interface governs the behavior of endogenous and artificial EPs [43]. A biomolecular protein corona forms rapidly when EPs are introduced into cell culture medium *in vitro* or plasma *in vivo* and can significantly affect nanoparticle physiology [44]. Context‐dependent EV and protein corona analysis, especially *in vivo* studies, are therefore highly relevant to gain translatable mechanistic insights into EV–cell interactions [44].

### 2.2 Extracellular vesicles in cancer

Cancer cell‐derived EVs have been described to carry divergent functional cargo ranging from genetic information, including DNA and RNA, to signaling proteins, lipids [3, 45, 46] and metabolites [47, 48]. EV cargo is regulated in a highly context‐dependent manner, for example, by cancer type (cell type) and its genetic and cell‐biological characteristics (cell state) [49]. Several excellent reviews focus on general vesicle biogenesis, cargo sorting, and EV release [1, 3, 50, 51].

Extracellular vesicles are released both from cancer cells and from cells of the tumor microenvironment, such as immune cells, mesenchymal stem cells (MSC), or fibroblasts [51]. EVs induce phenotypic changes in their target cells in both the local and distant tumor microenvironment and have been implicated in the establishment of a premetastatic niche and metastatic organotropism [52, 53, 54, 55, 56, 57]. EV‐mediated communication comprises tumor–tumor (autocrine and paracrine), tumor–stroma, and stroma–tumor communication. Considering their potential for systemic spread, for example, through the bloodstream, EVs also take part in cancer‐associated systemic events such as thrombosis [58], inflammation, and cancer immunity [12, 59].

To date, the biodistribution and organotropism of cancer‐associated EVs *in vivo* are still poorly understood. In particular, the proportion of unspecific, stochastic EV uptake versus specific uptake depending on EV physical characteristics, such as vesicle size and specific surface properties, remains currently unresolved [54].

## 3 Oncogenic and cancer‐associated signaling

By activating oncogenic signaling cascades, for example, through genetic events (loss‐of‐function mutations in tumor suppressors; or activating mutations in proto‐oncogenes) and epigenetic alterations, cancer cells evade physiological control mechanisms that stop uncontrollable cell growth [60, 61, 62]. In the early stages of tumor development, this oncogenic signaling activation is the driver of malignant transformation. Further dysregulations in signaling cascades that govern cell growth, division, motility, invasiveness, and cell death later drive tumor progression and metastasis. These cancer‐associated signaling cascades are not necessarily transforming, but nevertheless essential for the tumor to develop its full aggressiveness [60, 61, 62]. For example, signaling pathways in the tumor microenvironment coordinating angiogenesis, inflammation, or immune response affect tumor outgrowth and metastasis and can therefore be characterized as cancer‐associated signaling cascades [60, 61, 63].

Tumorigenic changes in cancer stem cell behavior, cell growth, survival, and proliferation often occur following the deregulation of signaling pathways, including the following: the wingless and Int/wingless‐related integration site (Wnt) signaling pathways (as, e.g., in colorectal cancer or breast cancer [64]); the transforming growth factor‐β (TGF‐β) signaling pathway (as, e.g., in colorectal cancer, gastric cancer, or endometrial cancer [65]); and the growth factor receptor epidermal growth factor (receptor) (EGFR)/human epidermal growth factor receptor (Her) signaling pathway (in epithelial tumors as, e.g., in squamous cell lung cancers, ovarian and breast cancer [66]). In addition, vascular endothelial growth factor (VEGF) signaling controls angiogenesis, and its dysregulation has been implicated in metastatic colorectal cancer, renal cell carcinoma, and non‐small‐cell lung cancer [67]. PD‐L1 overexpression as a result of altered cell signaling in cancer cells (such as melanoma and non‐small‐cell lung cancer) accelerates tumor progression by inactivating tumor‐specific T cells and mediating immune evasion [68].

The activation of oncogenes such as p53 or ras has been shown to disturb the cellular secretome inducing specific phenotypes, such as a VEGF‐mediated angiogenic [69, 70] or inflammatory phenotype [71]. Most likely the activation of oncogenic signaling cascades will therefore also influence EV secretion and EV‐mediated signaling—as it is described in the context of Wnt signaling [72]. At this point, the activation of an oncogenic signaling pathway can have an output that, when viewed individually, could be described as purely ‘cancer‐associated’. Nevertheless, if a signaling cascade is also described as transforming it is usually classified as oncogenic.

While EVs might play important roles also for other signaling pathways, in this review we focus on the current understanding on the role of EVs in the aforementioned signaling cascades. We discuss potential common mechanisms, including the transfer of pathway components, induction of EV release, therapeutic resistance, immune evasion, and possible clinical application.

## 4 EVs in Wnt signaling

**Fig. 2:**
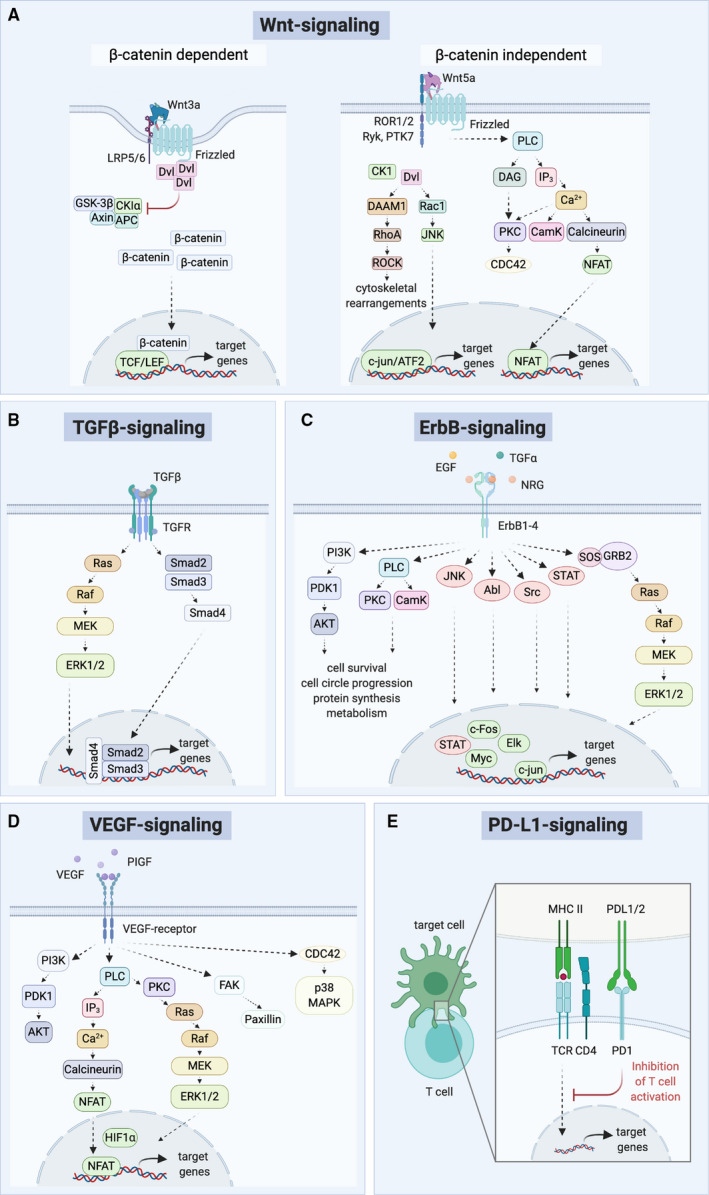
Overview of oncogenic signaling cascades discussed in this review. (A) β‐catenin‐dependent and β‐catenin‐independent Wnt signaling cascades. (B) TGF‐β signaling. (C) ErbB signaling. (D) VEGF signaling. (E) PD‐L1 signaling. Simplified representations.

The Wnt signaling pathways play essential roles during development, stem cell maintenance, and immune control. The oncogenic dysregulation of Wnt signaling has not only been linked to tumor initiation, but also to cancer progression and metastasis [64, 73, 74]. There are several major Wnt signaling branches: often characterized by their dependence or independence on β‐catenin and referred to as ‘canonical’ or ‘noncanonical’ pathways (Fig. 2A). Wnt pathways in mammals are activated by 1 of the 19 Wnt proteins, which bind to receptors of the Frizzled (Fzd) protein family and coreceptors. Depending on the Wnt–Fzd interactions and the involvement of coreceptors, such as LRP5/6, ROR1, ROR2, Ryk, or PTK7, different downstream signaling pathways are activated [75, 76]. In the case of the canonical Wnt signaling, which is β‐catenin‐dependent, β‐catenin is stabilized and translocates into the nucleus, where it induces together with other factors, such as TCF/LEF, the transcription of Wnt target genes. During β‐catenin‐independent signaling, multiple branches mediate transcriptional programs, as well as nontranscriptional outputs, such as cytoskeletal rearrangements [64, 74]. These modes of signaling can also have opposing effects and can be activated in a context‐dependent manner [64, 73, 74].

### 4.1 Wnt pathway components transported via EVs

Canonical Wnt ligands, such as Wnt3a, and noncanonical Wnts, such as Wnt5a, have been shown to be secreted on Exos and MV [77, 78]. It has further been shown that Wnt‐carrying Exos can act as signaling messengers, for example, in diffuse large B‐cell lymphoma [79], breast cancer [41, 78, 80], lung adenocarcinoma, colorectal cancer, and pancreatic cancer [81].

Recently, Scavo *et al*. [82] demonstrated that also the Wnt‐receptor Fzd10 travels via Exos and is thereby able to sustain and restore cancer cell proliferation of colorectal, gastric, hepatic, and bile duct cancer cells. Treatment of *FZD10*‐silenced cells with Exos of nonsilenced cells reinstated viability, and the Fzd10 protein and mRNA levels indicate its function for cancer reactivation and long‐distance metastasis [82]. In addition, increased plasma concentrations of Fzd10‐positive sEVs were associated with tumor progressions in colorectal and gastric cancer patients, suggesting that Fzd10 can serve as a biomarker for diagnostics and treatment response [83].

Furthermore, intracellular components of the Wnt signaling pathways, such as β‐catenin, have been found to be shuttled via EVs [84, 85, 86]. Chairoungdua *et al*. [84] showed that the tetraspanins CD82 and CD9 were necessary for the cellular export of β‐catenin via EVs. They showed an inhibitory effect of these tetraspanins in regulating Wnt/β‐catenin signaling by reducing the intracellular and nuclear β‐catenin levels [84]. Recently, Kalra *et al*. demonstrated that EVs transport mutant constitutively active β‐catenin [87], which is able to activate Wnt signaling in EV uptaking colorectal cancer cells. Using SILAC‐based quantitative proteomics, they also showed that mutant β‐catenin was recruited to the nucleus of the recipient cells to promote tumor progression [87].

### 4.2 Wnt signaling influences EV release

Interestingly, β‐catenin‐dependent and β‐catenin‐independent signaling components have been considered to interfere with mediators of EV release. In 2018, Lu *et al*. [88] identified multiple Wnt signaling and trafficking‐related genes in a genome‐wide miRNA and CRISPR/Cas9 screen as mediators of EV secretion. They demonstrated that Wnt‐mediated GSK3 inactivation regulated EV release by RAB27 mRNA and protein downregulation. The expression of RAB27B, which was activated by β‐catenin/TCF‐4, was also required for the release of Exos from colorectal cancer stem cells [89]. In patient‐derived colorectal cancer organoids, it was shown that the mutation of APC induces β‐catenin Wnt signaling, which resulted in an increased release of EVs [90].

β‐catenin‐independent Wnt signaling induces transcriptional and nontranscriptional events, such as cytoskeletal rearrangements, increased cell motility, and induced invasiveness [64], and multiple Wnt components influence factors of EV release such as Rabs, Rho‐GTPases, calcium influx, Arf6, and protrusion formation (mechanisms of EV secretion are reviewed in Ref. [1]). The noncanonical Wnt ligand Wnt5a has, for example, been shown in melanoma to induce the secretion of Exos enclosing the in this context immunomodulatory factor IL‐6 and proangiogenic VEGF and MMP2 [72]. This EV release was Ca^2+^‐dependent and could consequently be constrained by the calcium chelator Bapta [72]. Also, a dominant negative construct of the small Rho‐GTPase Cdc42 could prevent the Wnt5a‐induced EV secretion [72]. These findings gave further insights into basic EV secretory mechanisms and how Wnt signaling can influence it [72].

### 4.3 Wnt‐related EV cargo as a driver of tumor progression

Wnt signaling is a driver not only of tumor initiation but also of tumor progression and metastasis. In particular, the β‐catenin‐independent planar cell polarity pathway has been shown to promote cell migration and invasion [64]. Previously, EVs have been demonstrated to prepare the metastatic site for the arrival of circulating tumor cells and that they can induce malignant transformation in resident MSC [91]. In 2013, Lin *et al*. [92] showed that Exos of adipose tissue‐derived MSC can induce breast cancer cell migration by activating Wnt signaling pathways. Also in colorectal cancer, the delivery of Wnt1 via Exo(s) induced cancer cell migration [93]. The expression of long noncoding RNA (lncRNA)‐APC1 reduced colorectal cancer cell growth, metastasis, and neoangiogenesis by binding to RAB5b mRNA and inhibiting Exo production [93]. Other cells of the tumor stroma, such as macrophages [78] or cancer‐associated fibroblasts [41], are also able to promote tumor progression in a Wnt‐dependent, β‐catenin‐independent manner.

Neovascularization and angiogenesis are a prerequisite of growing tumors to sustain adequate supply of nutrients and oxygen and sufficient disposal of metabolic waste [61]. Exos of hypoxic colorectal cancer cells can promote angiogenesis by inducing the proliferation and migration of endothelial cells in a Rab27a/Wnt4/β‐catenin‐dependent way [92]. Wnt5a induction stimulated the release of proangiogenic Exo(s) from melanoma cells [72].

Cheng *et al*. [89] reported that Wnt‐dependent EV secretion induces an immunosuppressive microenvironment. β‐catenin/TCF‐4‐induced, secreted Exos showed miRNA‐146a‐5p (miR‐146a) as the predominant miRNA to target Numb, a protein crucial for asymmetric cell division during development, in recipient colorectal cancer cells promoting stemness and tumorigenicity [89]. In a cohort of 53 patients with colorectal tumors, patients with high levels of exosomal miR‐146a also displayed miR‐146a(high)/Numb(low) colorectal cancer stem cell traits, which correlated positively with the amounts of cancer‐infiltrating CD66 neutrophils and negatively with cancer‐infiltrating CD8 T cells [89].

To summarize, Wnt signaling and EVs have been tightly associated for many years [41, 77, 78]. Active Wnt proteins and other Wnt pathway components are transferred via EVs to mediate tumor initiation and progression, immune evasion, angiogenesis, and drug response. Recent findings discussed above also indicate that Wnt signaling induces a context‐dependent feedback mechanism of EV biogenesis and release.

## 5 EVs in TGF‐β signaling

The TGF‐β family members regulate cell fate during the early embryonic development and tissue homeostasis in adulthood [94]. Thirty‐three distinct mammalian genes of TGF‐β‐associated secretory factors include bone morphogenetic proteins and growth and differentiation factors [95]. These factors signal through heteromeric complexes of 12 kinase receptors, which can lead to the induction of SMAD‐dependent or SMAD‐independent pathways (Fig. 2B) [94]. The eight mammalian SMADs [referring to the homologies to *C. elegans* *Sma* (*small body size*) and drosophila *MAD* (*mothers against decapentaplegic*)] divide again into three functional groups: receptor‐regulated; inhibitory; or partnering Co‐SMADs. SMAD‐independent TGF‐β signaling involves, among other molecules, MAPK, JAK/STAT, Rho‐GTPases, and Akt [94]. TGF‐β can exert a myriad of cellular functions in a cell type and highly context‐dependent way [94]. In early stages of cancer and in benign cells, TGF‐β‐induced epithelial growth arrest is regarded as a tumor suppressor. Yet, TGF‐β cascades can contextually activate various oncogenic factors and in later stages of cancer have cancer‐associated signaling functions by stimulating epithelial‐to‐mesenchymal transition, promoting immune evasion, and inducing angiogenesis [96].

### 5.1 TGF‐β pathway components are transported via EVs

Cancer Exos are able to reprogram fibroblasts through TGF‐β on the surface of EVs inducing SMAD‐dependent signaling [97]. But also mast cell‐derived Exos were shown to carry active and latent TGF‐β1 on their surface to the endosome of receiving MSC [40]. At the time of signaling, TGF‐β1 was still in the endosomal compartment resulting in prolonged signal transmission compared with free TGF‐β1 [40]. Shelke *et al*. [40] also showed a migratory phenotype of the stem cells depending on SMAD activation. Goulet *et al*. [98] investigated the dedifferentiation of fibroblasts into cancer‐associated fibroblast by bladder cancer‐derived Exos and found that these Exos promoted invasiveness and metastasis. This process depended on SMAD activation in the cancer‐associated fibroblasts. It was also demonstrated that protumorigenic TGF‐β inside the EVs constituted 53.4–86.3% of TGF‐β in the supernatant [98].

Looking for further pathway components shuttled via EVs, also the active TGF‐β type II receptor has been described to shuttle between stromal fibroblasts and squamous carcinoma cells influencing TGF‐β signaling in the tumor–stroma interaction [99].

Additionally, Borzi *et al*. [100] showed that the oncoprotein c‐Myc delivered via tumor‐derived EVs can induce bronchial cell proliferation by overexpression of miR‐19b and miR‐92a and inhibition of TGF‐β. However, in pancreatic ductal adenocarcinoma Yin *et al*. [101] reported the relevance of M2 macrophage‐derived exosomal miR‐501‐3p to inhibit the tumor suppressor TGFBR3 and therefore facilitating tumor development by TGF‐β signaling leaving question of context dependencies not only of EV‐mediated signaling but also of TGF‐β signaling *per se*.

### 5.2 TGF‐β‐dependent alterations of EV cargo

Microsatellite instable colorectal cancers frequently harbor inactivating mutations of the TGF‐β receptor type 2 (TGFBR2) [102]. Studies of the protein and miRNA expression profiles of EVs from TGFBR2‐deficient and microsatellite instable colorectal cancers revealed an upregulation of certain ECM and nucleosomal proteins and a downregulation of proteasomal proteins in the EVs of TGFBR2‐deficient cancers [102]. Fricke *et al*. [102] characterized the impact of TGFBR2 alterations on the miRNA profiles of parental cells and EVs using small RNA sequencing. In a similar way, Kang *et al*. [103] demonstrated an induction of tumor‐promoting PD‐L1 by TGF‐β via SMAD2/3 and YAP/Taz in murine and human fibroblasts. PD‐L1‐carrying EVs were able to inhibit T‐cell proliferation and mediate fibroblast cell migration/wound healing [103].

### 5.3 TGF‐β‐related EV cargo as a driver of tumor progression

Implementing an immunosuppressive microenvironment supports undisturbed tumor outgrowth and metastasis of tumors. Several studies indicate that TGF‐β‐bearing EVs are involved in the communication of cancer and immune cells.

In 2011, Szczepanski *et al*. [104] showed that patient‐derived MV from acute myeloid leukemia blasts were able to suppress natural killer cell functions through membranous TGF‐β1. Recently, Zhao *et al*. [105] investigated the role of natural killer cells in the immunosuppressive effects in the pancreatic cancer environment. Quantification of serum EV‐TGF‐β1 content by ELISA showed a significant increase in pancreatic cancer patients, while coculture experiments of EVs with natural killer cells demonstrated a significant downregulation of various immunomodulatory proteins (such as NKG2D, CD107a, TNF‐α, and INF‐γ) [105].

Tumor Exos also induce myeloid‐derived immune cells promoting tumor progression through prostaglandin E2 and TGF‐β [106]. Umakoshi *et al*. have reported a similar role for macrophages in the EV–stroma interaction by transferring cancer‐derived components such as TGF‐β and Wnt3 via EVs and establishing a protumorigenic microenvironment in gastric cancer [107]. Rong *et al*. [108] showed suppression of T‐ cell proliferation by hypoxia‐induced, breast cancer, and exosomal TGF‐β. In melanoma, the major histocompatibility complex proteins were transferred to the surface of antigen‐presenting cells to induce costimulatory immune receptors, to upregulate IL‐6, and to transfer TGF‐β [109].

For premetastatic niche formation in distant organs, Costa‐Silva *et al*. showed not only the above‐mentioned central role of sEVs from pancreatic cancer cells, they also point out a role of TGF‐β signaling activation leading to ECM remodeling, fibronectin induction, and the influx of bone marrow‐derived macrophages to the liver, which are favorable for consecutive liver metastasis [52].

In the last years, TGF‐β signaling and EVs have been linked to cancer especially in the communication of tumor and stroma cells. Since TGF‐β was described as an oncogene and a tumor suppressor in a highly context‐dependent way, it might be challenging to apply EVs as universal prognostic tumor markers. However, the role of EV‐TGF‐β signaling for the induction of an immunosuppressive tumor environment has been well established. This opens up possibilities for combinatorial treatment schemes, including approaches to regenerate antitumor immunity.

## 6 EVs in ErbB signaling (EGFR/HER)

The ErbB family of receptor tyrosine kinases comprises four family members ErbB1, 2, 3, and 4 (also Her1‐4). ErbB1 is the best described one and commonly known as epidermal growth factor receptor (EGFR) [66]. Binding of a ligand such as TGF‐α or EGF initiates homo‐ or heterodimerization of ErbB receptors, which in turn results in the activation of downstream factors including PI3K‐Akt‐Tor, Ras‐Raf‐MEK‐ERK, STAT and protein kinase C (Fig. 2C) [110].

Oncogenic ErbB signaling is crucial for cell growth and inhibition of apoptotic cell death in development and disease [66]. Overactivation of various signaling branches, for example, EGFR and HER, has been associated with the development of epithelial malignancies promoting tumor growth, invasion, and metastasis [111].

In adult normal tissues, ErbB ligands and the expression of the respective receptors are tightly regulated. By contrast, in tumor tissues the production of ligands can be sustained by the tumor microenvironment and the receptors are frequently overexpressed and/or mutated rendering them as important therapeutic targets [111].

### 6.1 ErbB pathway components are transported via EVs

Al‐Nedawi *et al*. reported the transfer of the truncated and oncogenic EGFRvIII by brain tumor cell MV [21], a result confirmed and extended by Skog *et al*. later that year [112]. They showed tumor‐promoting capacities of RNA and protein containing glioblastoma MV [112]. In particular, the tumor‐specific EGFRvIII was present in serum EVs [112].

Later, it was shown that Exos from gastric cancer cells were able to transfer active EGFR complexes to stromal liver cells where they were integrated into the outer plasma membrane [113]. Thus, the translocated EGFR activated hepatocyte growth factor by miR‐26a/b suppression helping to establish the tumor‐niche and liver‐specific metastasis [113].

Interestingly, Read *et al*. [114] described an additional mechanism, wherein EV‐EGFR and EV‐EGFRvIII were transported into the nucleus of EV‐receiving cells independent of EGFR’s nuclear localization sequence. Nevertheless, EGFR was still capable of activating downstream pathways [114].

A recent in‐depth analysis of *in vitro‐* and *in vivo*‐released EV subpopulations from glioblastoma cells expressing the *mutant EGFR U87^EGFRvIII^* showed that that small and large EVs contained tumor‐specific EGFRvIII mRNA and proteins and that the EGFR protein content (wild‐type and mutant) was significantly higher in the latter [115].

### 6.2 ErbB pathway‐dependent alterations of EV cargo

Analysis of the EV proteome of a mammary luminal epithelial cell line (HB4a) and a HER2‐overexpressing variant demonstrated that not only HER2 signaling (sphingosine‐1‐phosphate pathway)‐associated proteins, but also proteins controlling cell motility and invasiveness were differentially expressed in the respective EVs indicating their malignancy and their potential function as biomarkers [116].

Looking at ErbB1, Choi *et al*. [117] investigated the influence of mutated EGFRvIII on the proteomic profile of EVs released from Glioblastoma cells. EVs from EGFRvIII‐expressing cells expressed increased homophilic adhesion molecules and homologous uptake by EGFRvIII glioma cells [117].

Montermini *et al*. [118] reported a connection of EGFR inhibition and the release of EVs with changed phosphoprotein and DNA content. They showed that phosphorylated EGFR (P‐EGFR) among other receptor kinases could be found in plasma EVs of mice with malignant tumors [118]. Interestingly and in contrast to cetuximab (anti‐EGFR antibody) or etoposide (topoisomerase inhibitor inducing apoptosis independently of EGFR), the administration of kinase inhibitors, such as CI‐1033 and PE‐00299804, triggered the release of EVs containing varying profiles of (P‐)EGFR and genomic DNA [118]. Treatments with GW4869 (a selective inhibitor of the neutral sphingomyelinase and thereby EV generation) and ZVAD (caspase pathways inhibitor) were capable of attenuating this induced EV release[118]. The group could therefore demonstrate how targeted agents are able to change EV profiles and EV shedding in response to therapeutic stress [118].

### 6.3 ErbB pathway‐related EV cargo as a driver of tumor progression

Microvesicles containing the truncated and oncogenic EGFRvIII were shown to fuse with the plasma membrane of EGFRvIII‐negative tumor cells, inducing the oncogenic phenotype via horizontal transfer and activating oncogenic signaling via MAPK and AKT [21]. Later, Al‐Nedawi *et al*. also demonstrated a proangiogenic effect of EGFR‐positive MV on endothelial cells through induction of VEGF expression and autocrine VEGFR‐2 activation [119].

The impact of mutated, exosomal EGFR (EGFR E746‐A750 Deletion/EGFR‐19del) on the antitumor immune response of lung cancer and its relevance for drug response was recently reported by Yu *et al*. [120]. Patients with EGFR‐mutated lung cancer responded only poorly to PD‐1/PD‐L1 blockage, and Yu *et al*. [120] found an association of the EGFR‐19del mutation with reduced numbers of intratumoral CD8^+^ T cells at early disease stages. Surprisingly, dendritic cells carrying the EGFR‐19del mutation were detected within lung tumors, in both mouse and human samples. *In vitro* experiments indicated exosomal shuttling of mutated EGFR from lung cancer cells into dendritic cells [120]. The integration of these active but mutated receptors into the dendritic cell surface promoted tumor progression and induced immunosuppression [120]. The combinatorial treatment with gefitinib, a kinase inhibitor commonly used in bronchial carcinoma, and granulocyte–macrophage colony‐stimulating factor rescued dendritic cell function and restored T‐cell infiltration in EGFR‐19del tumors increasing subsequently the effectiveness of anti‐PD‐L1 checkpoint inhibitors [120].

In summary, the early functional analyses of ErbB signaling via EVs broadened our understanding of the complexity of EV‐mediated signaling. Not only ligands, but also active receptor complexes can be transferred via EVs and can be shuttled to various intracellular compartments. Additionally, EVs carrying (mutated) EGFR/HER2 were established as potential biomarkers in liquid biopsies from cancer patients for disease diagnosis or the prediction of therapeutic response. Lastly, research in the ErbB signaling field tested in various settings the targeted delivery of therapeutics via EVs to only a subpopulation of receptor‐expressing cells (see also Section 9 on diagnostic and therapeutic applications of EVs).

## 7 EVs in VEGF signaling

The VEGF signaling pathway regulates the development of blood and lymphatic vessels in physiological and pathological processes during development and adulthood, also in tumor progression and metastasis [121]. This cancer‐associated signaling pathway is activated by binding of one of the five mammalian VEGF ligands (A‐D and placental growth factor, PIGF) to one of three receptor tyrosine kinases (VEGFR 1‐3) (Fig. 2D) [121]. The recruitment of coreceptors, such as neuropilins and integrins, can modulate the signaling outcome [121]. VEGF and its receptors are frequently found to be overexpressed in tumors [122]. Hence, targeting antibodies and kinase inhibitors impairing tumor growth have been of great research interest at the bench and bedside in the last years [122].

### 7.1 VEGF pathway components are transported via EVs

Treps *et al*. [123] showed in 2017 that glioblastoma cells with stem‐like properties are able to secrete active VEGF‐A and mediate tumor‐induced angiogenesis by utilizing EVs.

Conley *et al*. [124] performed high‐throughput sequencing of EVs isolated from the peripheral blood of patients with breast cancer, and identified a tumor‐specific mRNA signature in large oncosomes versus Exos. VEGF‐A mRNA appeared enriched in large oncosomes, but the biological implications of this finding remain elusive [124].

### 7.2 VEGF‐related EV cargo as a driver of tumor progression

Changes in the cellular microenvironment, such as pH alterations induced by cancer therapeutics or other conditions, can lead to the induction of EV shedding [42, 125]. But the initial observation of *in vitro* EV shedding after treatment with certain drugs led to broad discussions about whether these EVs are apoptotic bodies from dying cells or EVs originating from living cells. The signaling activity of these EVs has also been disputed [8, 126].

Recently, Vera *et al*. [127] demonstrated that cancer therapy promoted protumorigenic EV cargo. They showed that ovarian cancer spheroids released sEVs in response to cisplatin. These EVs in turn induced a migratory phenotype in bone marrow‐derived MSC by increasing gene expression of metalloproteinases [127]. This phenotype was accompanied by increased secretion of interleukins (IL‐6 and IL‐8) and VEGF‐A from MSC resulting in the inducing of angiogenesis in endothelial cells [127]. Altogether, these data suggest that actively secreted EVs participating in the tumor–stroma interaction can contribute to the adverse effect of cancer therapy [127].

Recent work of Wang *et al*. [128] gave insights into the role of EVs in the lymphovascular invasion and the early dissemination of pancreatic ductal adenocarcinoma. They showed that the suppression of dual‐specificity phosphatase‐2 enhanced a proprotein convertase activity via ERK1/2. This in turn increased the secretion of EVs carrying VEGF‐C inducing lymphangiogenesis and lymphovascular invasion [128].

Vascular endothelial growth factor signaling is one of the main drivers of neoangiogenesis, which is one of the hallmarks of cancer [61, 62]. Deeper understanding of the mechanisms how VEGF‐carrying EVs mediate their effects on the receiving cells are a prerequisite to further harness them for therapeutic strategies (see also Section 9 on diagnostic and therapeutic applications of EVs).

## 8 EVs and regulation of the PD1–PD‐L1 pathway

Programmed cell death 1 ligand 1 (PD‐L1, also known as CD274 or B7‐H1) is a type I transmembrane protein that binds to the programmed cell death protein 1 (PD‐1) on immune cells to inhibit their and prevent autoimmune reactions (Fig. 2E) [129]. Many tumors upregulate the expression of PD‐L1 to escape recognition by the immune system, thereby promoting tumor progression [129]. Therapies modulating immune checkpoints emerged as powerful treatment options for various cancers, such as melanoma and lung cancer, resulting in increased patient survival [68, 130].

Initial or acquired resistance to PD‐L1‐directed therapeutics harbors a great burden for patients with the above‐mentioned malignancies, since they cannot benefit from these treatment options [131]. Early predictions of treatment response could protect these patients from potential adverse events of therapies with no clinical benefit.

### 8.1 PD‐L1 is transported via EVs

The presence of PD‐L1 on the surface of EVs has been recently described not only for various solid tumors, such as breast cancer [132], metastatic melanoma [133], glioblastoma [134], head and neck squamous cell carcinoma [135], and gastric [136] and pancreatic cancer [137], but also for chronic lymphocytic leukemia [138].

Interestingly, PD‐1 was not detected on Exos derived from chimeric antigen receptor (CAR) T cells, although the maternal cells expressed the immunosuppressive receptor [139]. In their study, Fu *et al*. [139] compared the antitumor effects and toxicity of effector CAR T‐cell‐derived Exos with the application of CAR T cells only. CAR Exos carried high amounts of cytotoxic cargo and therefore were still able to inhibit tumor growth [139]. And since they did not carry PD‐1, their antitumor effect would most likely not be diminished by recombinant PD‐L1 treatment [139].

### 8.2 PD‐L1‐positive EVs as drivers of tumor progression

Since immune evasion is one of the major drivers of tumor progression, EVs carrying PD‐L1 and their effect on various immune cells has been extensively investigated in the last years.

Stimulation with interferon‐γ increased the levels of PD‐L1 on Exos and suppressed T‐cell function in the melanoma tumor environment [133]. In glioblastoma, defined by local and systemic immunosuppression, PD‐L1 was expressed on a subset of tumor EVs and inhibited T‐cell activation in a PD1‐dependent manner [134]. The blocking of PD1 with antibodies significantly reduced the EV‐mediated T‐cell suppression and prevented tumor progression [134]. Nevertheless, no significant improvement in patient survival could be achieved by immune checkpoint inhibitors in patients with glioblastoma [140]. T‐cell suppression by exosomal PD‐L1 was also reported in the context of breast cancer [132].

Focusing on myeloid‐derived cell lines, Ning *et al*. [141] showed that tumor Exos have an effect on the maturation of dendritic cells. Tumor EVs impaired dendritic cell maturation, thereby promoting T‐cell suppression, and this effect was partially reversible by blocking of PD‐L1 [141]. Fleming *et al*. [142] investigated how tumor EVs can transform myeloid cells into myeloid‐derived suppressor cells. Melanoma cell‐derived EVs were able to upregulate PD‐L1 via activation of TLR4 signaling and subsequently induce immunosuppressive monocytes in a HSP86‐dependent manner [142]. Exosomal transfer of the noncoding Y RNA hY4 to monocytes led to upregulation of PD‐L1 on these cells, thereby contributing to cancer‐associated inflammation and immune escape in chronic lymphoid leukemia [138].

The effect of exosomal PD‐L1 on immune evasion was also shown in genetic murine models [143]. Here, PD‐L1 suppression did not only induce antitumor immunity at the primary tumor site but also induced a systemic immune response [143].

### 8.3 PD‐L1 signaling and EV release

Changes in the microenvironment can interfere with EV release, such as changes in pH [42, 133], thermal and oxidative stress [144], and hypoxia [145]. Therefore, it is not surprising that chemotherapy and radiotherapy have been associated with the release of different populations of EVs [146]. Radiotherapy is additionally known to induce various immune responses in cancer patients [146]. Radiotherapy‐induced microparticles from breast cancer cells carry cargo containing distinct immunomodulatory proteins, among those also PD‐L1 that suppressed T‐cell function and promoted tumor growth. Accordingly, a therapeutic synergy of radiotherapy and immune checkpoint modulators has been proposed [146].

With the fast developing field of research on PD‐L1 and immune checkpoint modulation, PD‐L1 on EVs arose as potent mediators of immune evasion. At the same time, PD‐L1‐bearing EVs were successfully tested for the prediction of treatment response in various tumors (see also Section 9 on diagnostic and therapeutic applications of EVs).

**Fig. 3:**
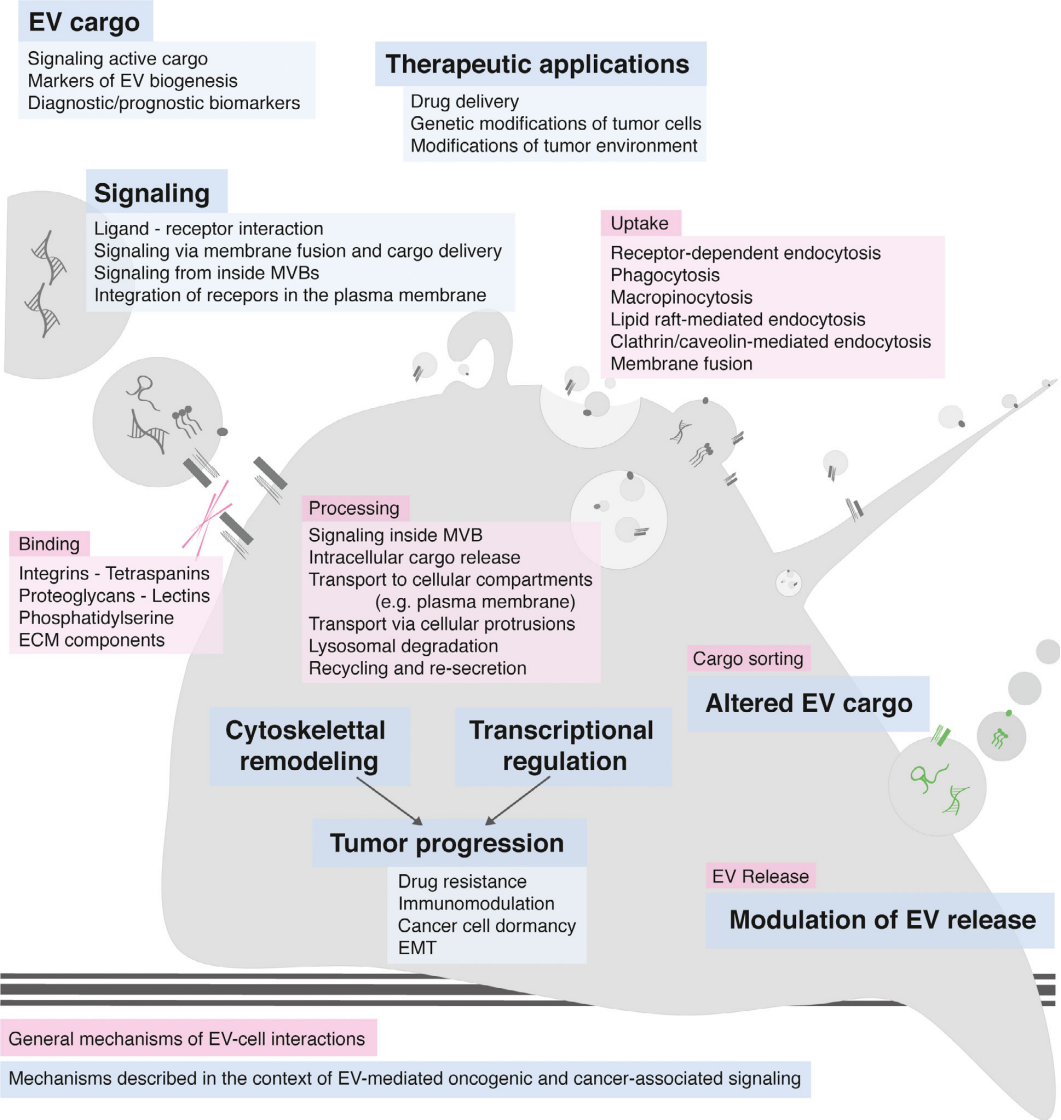
EV–cell interaction. General mechanisms of EV–cell interaction, such as EV binding, uptake, processing, cargo sorting, and EV release, are depicted in pink. Specific mechanisms and effects of EV–cell interaction described in the context of oncogenic signaling cascades are highlighted in blue. Components of oncogenic and cancer‐associated signaling cascades have been described as EV cargo, inducing intracellular signaling through various mechanisms, influencing tumor progression through transcription‐dependent and transcription‐independent events, altering EV cargo and modulating EV release.

**Fig. 4:**
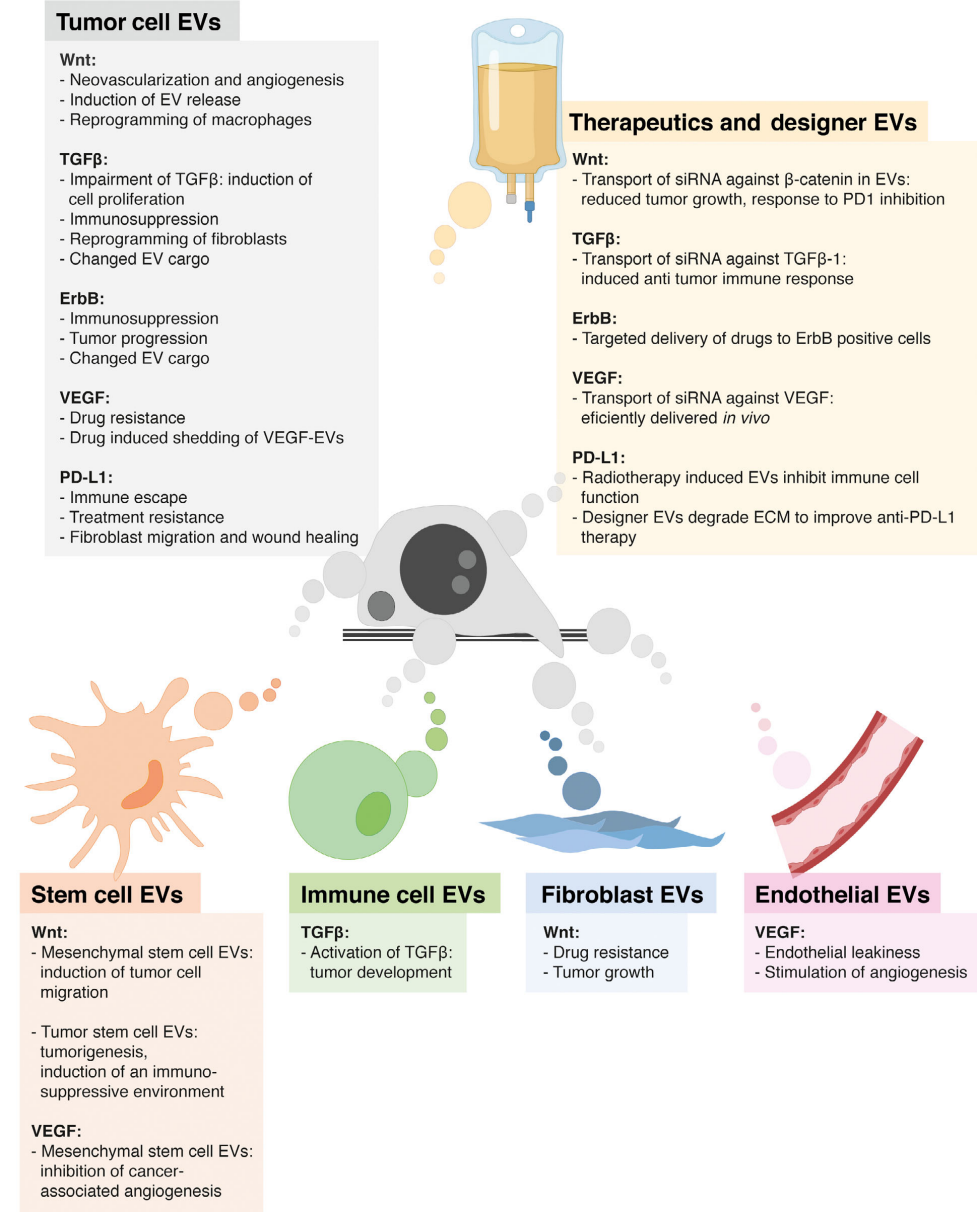
Multifaceted effects of EVs in oncogenic and cancer‐associated signaling. Illustrative summary of how EVs from different origins use the indicated signaling pathways to drive cancer initiation, progression, and metastasis. As examples, tumor cell EVs influence neovascularization and angiogenesis through activation of Wnt signaling; radiotherapy‐induced EVs are able to inhibit immune cell function via PD1–PD‐L1.

## 9 Diagnostic and therapeutic applications of EVs

### 9.1 EVs as biomarkers

In particular in colorectal cancer, where Wnt signaling has been established as one of the drivers of tumorigenesis [64] and the role of EVs in mediating this Wnt‐response has been substantiated, EVs have been proposed as biomarkers [93]. But also in breast cancer, Wnt10b‐positive Exos were suggested as prognostic markers governing breast cancer cell metastasis [80].

In the context of TGF‐β signaling, Rodrigues *et al*. [147] suggest to use EVs as *bona fide* biomarkers in the clinical routine. They tested them for noninvasive monitoring of therapeutic response to chemoradiation therapy. TGF‐β3 protein levels were found significantly higher in locally advanced head and neck squamous cell carcinoma patients treated with chemoradiation therapy [147]. Additionally, they demonstrated TGF‐β3 silencing sensitized cancer cells toward cytotoxic therapy and that the resistant phenotype can be mediated via EVs suggesting a sensitization approach using TGF‐βR inhibitors [147].

Epidermal growth factor (receptor) amplifications and EGFRvIII mutations are commonly detected in various cancer patients [148]. Patients with glioblastoma multiforme release EVs from the tumor into the cerebrospinal fluid (CSF). The comparison of wtEGFR and EGFRvIII RNA expression in CSF‐derived EVs and in matching tumor tissues from patients undergoing tumor resections showed that EV RNA signatures mirrored the genetic status of the tumors [148]. These results suggest a less invasive diagnostic option to direct therapeutic strategies in the future [148].

Extracellular vesicles carrying PD‐L1 might be useful as diagnostic and prognostic markers, for example in pancreatic cancer [137], and as indicators of treatment response as reported in the context of head and neck squamous cell carcinoma [135], non‐small‐cell lung cancer [149], and melanoma [133, 149]. The level of increase in the PD‐L1‐carrying Exos in the early stages of cancer treatment was able to stratify responders from nonresponders turning exosomal PD‐L1 into a predictor of drug resistance and therefore tumor progression [133]. Accordingly, a prospective study by Cordonnier *et al*. [150] established high PD‐L1 levels of circulating Exos as an even more reliable predictor of treatment response than PD‐L1 expression in melanoma biopsies. In another study, Exo amounts and markers of patients with head and neck squamous cell carcinoma were characterized in response to treatment with ipilimumab (monoclonal antibody targeting the immune checkpoint CTLA‐4), cetuximab (anti‐EGFR antibody), and radiation therapy enrolled in a phase I clinical trial [151]. The comparison of relapsed versus recurrence‐free patients revealed decreased tumor Exos (total exosomal protein and tumor cell Exo levels) in the latter [151]. In contrast, recurrence was associated with increased total Exo protein, an increment of tumor exosome‐to‐total exosome ratio, and total CD3^+^, CD3^‐^ PD‐L1^+^, and CD3^+^ 15s^+^ (Treg‐derived) Exos [151].

Despite these recent advances in the search for diagnostic and prognostic markers, none of the above‐mentioned EV‐associated biomarkers have been approved by national or international agencies controlling the approval of new biomedical test and testing devices, such as the U.S. Food and Drug Administration (FDA). Nevertheless, for the detection of EV‐associated biomarkers in prostate cancer progress can be noted, since recently ExoDx Prostate (IntelliScore) (EPI) [152, 153] was the first approved device for Exo‐based liquid biopsy by the FDA.

### 9.2 EVs and their influence on drug response

Cancer stem cells harbor certain characteristics such as chemotherapy resistance [154]. Accordingly, cancer stem cells are enriched in tumors where therapeutic regimens failed [155]. In the context of Wnt signaling, Hu *et al*. [156] described dedifferentiation of colorectal cells by fibroblast‐derived Exos promoting chemoresistance. Inhibiting Wnt release via these EVs diminished this effect *in vitro* and *in vivo*. Furthermore, it was shown that cancer‐associated fibroblasts are able to promote drug resistance and stemness, epithelial–mesenchymal transition (EMT), and metastasis of colorectal cancer cells through Exos increasing miR‐92a‐3p levels [156]. MiR‐92a‐3p‐activated β‐catenin‐dependent Wnt pathways inhibited mitochondrial apoptosis [156]. In addition, miR‐92a‐3p expression was significantly increased in tissue samples from colorectal cancer patients and high exosomal miR‐92a‐3p of serum was correlated with resistance to chemotherapy and metastasis in patients with colorectal cancer [156]. These data propose miR‐92a‐3p as an indicator of therapeutic response and a potential target for the treatment of metastasis in colorectal cancer [156].

In the context of ErbB signaling, drug resistance to trastuzumab (anti‐HER2 humanized monoclonal antibody) has been proposed by the transfer of lncRNAs [157, 158]. Recently, they found that exosomal lncRNA AFAP1‐AS1 promoted drug resistance by binding to RNA‐binding factor‐1 (AUF1), which activated ERBB2 translation [158].

For VEGF signaling, another mechanism of EVs mediating drug resistance has been described by Feng *et al*. [159] A VEGF variant (VEGF90K) on MV not only activated VEGF receptors, but also showed a weaker affinity to the humanized anti‐VEGF monoclonal antibody bevacizumab. This caused an ineffective inhibition of the VEGF receptor activation by bevacizumab [159]. Ko *et al*. [160] showed a similar mechanism for VEGF189 that preferentially localizes to sEVs through its high heparin binding capacities. Through this, VEGF’s half‐life was prolonged and binding to bevacizumab was inhibited. They also found a correlation of the amounts of sEV‐VEGF with disease progression of patients under bevacizumab treatment, claiming it to be a potential marker for treatment response [160]. Nevertheless, more detailed and mechanistic comparison of the resistance not only to bevacizumab but also to membrane‐permeable receptor tyrosine kinase inhibitors targeting VEGF (e.g., sunitinib) could give further insights not only into EV‐resistance mechanisms but also into tumor biology and primary resistance to receptor tyrosine kinase inhibition.

Likewise, EGFR/Her1 and HER2 have been found on EVs from the cancer cell lines SkBr2 and BT474 that both overexpress HER2 [161]. Interestingly, the secretion of these EVs was regulated by heregulin and EGF*—*two ligands activating HER2 signaling [161]. Xenogeneic and autologous HER2‐positive EVs inhibited the antiproliferative effect of trastuzumab, whereas lapatinib activity (EGFR tyrosine kinase inhibitor) was not affected [161].

This neutralizing effect on commonly applied, therapeutic monoclonal antibodies by epitope presentation on EVs most likely constitutes a basic mechanism of altered drug response that can possibly be transferred into multiple other signaling contexts.

As stated above, also PD‐L1‐positive EVs can be used as biomarkers for treatment resistance. Nevertheless, the question how exosomal PD‐L1 mediates resistance to anti‐PD‐L1 therapy *in vivo* is still debated [143]. Possible reasons are discussed by Poggio *et al*. who state most likely transferable mechanisms to be considered in other contexts: First of all, the presentation on EVs itself might hinder the recognition of PD‐L1 by common antibodies [143]. Secondly, the amount of PD‐L1 when also found on Exos might be at drastically higher levels, so that the delivered quantities of antibody are insufficient [143]. Thirdly, Exos might access areas that cannot be reached by antibodies protecting them from inactivation [143]. Taken together, the crosstalk of EV surface antigens with respective antibodies requires further context‐dependent analyses to gain mechanistic insights.

### 9.3 Therapeutic applications of EVs

Wnt/β‐catenin signaling activation can contribute to immune evasion [64, 162]. Matsuda *et al*. [163] investigated the feasibility of an EV‐based therapeutic targeting of β‐catenin in hepatocellular cancer. Aiming to enhance the therapeutic response to other immunomodulatory drugs, such as checkpoint modulators such as PD1 antibodies, they showed in a transgenic mouse model of hepatocellular carcinoma the efficacy of targeting β‐catenin using siRNA‐carrying EVs [163]. They therefore effectively reduced tumor growth and enhanced the therapeutic response to PD1 inhibition [163]. Reducing systemic adverse events by delivering therapeutic agents directly to the target site via EVs could be a potential strategy for targeting Wnt signaling in cancer.

In the context of TGF‐β signaling, Rossowska *et al*. [164] tested the potential of genetically engineered EVs from MC38 colon carcinoma cell lines as tools for antitumoral therapy in mice. They designed EVs overexpressing IL‐10 and/or shRNA for TGF‐β1 for application as single treatments or in combination with dendritic cell‐based vaccines in mice with subcutaneous MC38 tumors [164]. The designer EVs were able to inhibit tumor growth and regenerate antitumor immunity accompanied by a significant increase in T helper type 1 cell response in the combinatorial treatment approach [164]. These data suggest that EVs loaded with IL‐12 or shRNA for TGF‐β1 can be applied in an adjuvant setting in immune and chemotherapeutic treatment schemes to induce an antitumor immune response [164]. Results from Huang *et al*. had suggested similar effects in the context of antileukemia immunity by leukemia‐derived Exos and reduced TGF‐β1 expression [165].

For ErbB signaling, Ohno *et* *al*. [166] demonstrated in 2013 that engineered, systemically injected Exos were able to target specifically EGFR‐expressing breast cancer cells thereby effectively delivering microRNAs. Hijacking this EGFR‐specific EV uptake, Kooijmans *et al*. [167] engineered glycosylphosphatidylinositol (GPI)‐anchored anti‐EGFR nanobodies on the EV surface to promote tumor cell‐specific targeting. Also, efficient and specific delivery of doxorubicin (cytotoxic anthracycline) to HER2^+^ breast cancer cells was made possible by loading Exos and expressing a chimeric LAMP2b‐DARPin protein on the surface of EVs [168]. Gomari *et al*. [168] additionally reported a significant reduction in tumor growth by the administration of targeted Exos but not of free or untargeted‐exosomal doxorubicin in a murine breast cancer model. Wang and Forterre and colleagues investigated in the context of HER2^+^ breast cancer feasible and safe administration options to specifically guide prodrug/enzyme regimens to cancer cells with minimal off‐target toxicity [169, 170]. They also used EVs for specific targeting of only the HER2^+^ subpopulation through a chimeric protein designed to be presented at the EV surface. Therefore, they successfully administered *in vitro*‐transcribed mRNA through EVs [169, 170].

In the future, these approaches will have to be tested in more clinically relevant model systems (e.g., using autologous EVs or immunocompetent animals) to ensure lasting immunological tolerance and to investigate their therapeutic efficacy and feasibility to enable translation into the clinical settings. Also, the transfer of these established tools and methodologies into other pathological contexts, where other signaling cascades are involved, especially in pathologies currently lacking therapeutic options, will be fundamental.

One of the benefits of using EVs as carriers for therapeutic applications is their biological properties, such as a potentially prolonged half‐life time and their ability to cross biological borders, such as the blood–brain barrier [16]. *In vivo* studies in zebrafish by Yang *et al*. showed the delivery of exogenous siRNA by brain endothelial cell‐derived Exos inhibiting VEGF‐ signaling [171].

In a slightly different but highly innovative approach, Hong *et al*. used EVs to modify the tumor microenvironment [172]. They tested the application of GPI‐anchored sEV‐PH20 hyaluronidase to penetrate the tumor through hyaluronan (HA) degradation [172]. Highly accumulated HA serves as an immune‐suppressive barrier in the tumor environment, whereas oligo‐HA molecules stemming from HA degradation act as Toll‐like receptor‐4 agonists leading to the activation of CD103^+^ dendritic cells and subsequently tumor‐specific CD8^+^ T cells [172]. In a murine breast cancer model, the combined treatment of GPI‐anchored sEV‐PH20 hyaluronidase together with an anti‐PD‐L1 antibody had a more potent tumor suppressive effect than either monotherapy arguing that engineered Exo‐PH20 could serve as a potential agent for immunological cold tumors [172].

The therapeutic application of MSC‐derived EVs exploiting their multiple beneficial properties has been the focus of many researchers in the last years: In 2013, Lee *et al*. [173] demonstrated that MSC‐derived Exos were able to reprogram the breast cancer tumor microenvironment by transferring their molecular cargo. The downregulation of VEGF expression in tumor cells, at least partially by transfer of miRNA‐16, reduced angiogenesis *in vitro* and *in vivo* [173]. The study indicates a great potential of MSC‐derived EVs to change, for example, the vascular behavior in the microenvironment, identifying them as a potential tool to target cancer‐associated angiogenesis [173]. For comprehensive graphical summaries of EV–cell interactions and the multifaceted effects of EVs from different origins in the context of cancer‐associated signaling please see Figs. 3 and 4, respectively.

## 10 Outlook

Signaling via EVs during tumor initiation, progression, and metastasis has become an important research area with implications for the analysis of liquid biopsies, cancer diagnostics, prognostics, and therapeutic approaches. To gain a deeper and mechanistic understanding of signaling components present in or on EVs will be one of the major challenges in the next years for correct translation into the clinical setting.

Unfortunately, further oncogenic and cancer‐associated signaling cascades could not be covered in this review. However, this does not mean that they have no role in the EV–cell communication. Indicatively, oncogenic NOTCH signaling and hormone receptor‐mediated signaling are further highly relevant examples of EV‐mediated signaling cascades in the tumor–stroma interaction.

To exploit EVs for early cancer detection or to interfere with EV biogenesis, spread, or signaling, it is crucial to find answers to the following questions: Which cargo is found in or on EVs and what is its role in EV biogenesis? Which cargo of EVs has functional implications and which is ‘hitch‐hiking’ because of its expression in the cell lineage or tumor context? To find answers, it will be necessary to establish readouts in order to demonstrate the specific activation of a signaling pathway by the respective signaling agent (e.g., transcriptional assays, clear phenotypic changes). Current studies often correlate EV uptake with a certain, rather unspecific outcome failing to answer questions of exact biological mechanisms of signaling induction [174]. Analyses are needed that investigate further EV processing such as integration into the plasma membrane, EV/cargo degradation, endosomal escape, or EV recycling and resecretion [175].

Technical improvements will be a prerequisite [2, 175]. Lack of sensitivity and specificity of commonly used methods and available materials hinders in‐depth EV research, for example, due to low sensitivity or lack of specificity of commonly used antibodies [2]. Additionally, image‐based analysis is limited not only by the diffraction limit but also by detection levels, particularly when investigating rare and/or endogenous levels of proteins. Furthermore, we are in need of methods allowing the analysis of EVs independent of isolation steps and processing, which always need to be suspected for affecting EV characteristics and signaling behavior [175]. Contaminations of EV preparations with additional soluble factors should be thoroughly ruled out. The suggested biological relevance of highly abundant soluble versus potentially lowly abundant EV‐bound factors should critically be questioned. Last but not least, there is great need for reliable protocols for EV tracking *in vivo* [174, 175]. Until then, the knowledge of the limitations of the applied methods is essential for correct data analysis and interpretation. Not only the critical analysis of the power of the applied experimental setups, but also continuous technical improvements are essential to boost the quality of EV research and bring about benefit for cancer diagnostics and therapeutic advances.

Autologous EVs are characterized by low immunogenicity and therefore remarkable biocompatibility [176]. This increases circulatory capabilities and supports the passing of biological borders such as the blood–brain barrier [16] rendering them as interesting vehicles for therapeutic approaches [176]. But for a therapeutic application of EVs, clear definitions of EV subtypes are indispensable to provide comprehensive and comparable analyses in the future [2]. Furthermore, the cultivation of the cell lines, large‐scale isolation protocols, in‐depth EV characterization, storage, and application need to be standardized [177]. *In vivo* studies will also help to better understand the challenges of EV‐based therapeutic approaches, such as EV half‐life time and unspecific or limited targeting. Engineering methods to incorporate various cargoes into EVs has been proven successful, and phase I clinical trials have tested the feasibility of large‐scale EV production and safety in patients with various cancers (as reviewed in Ref. [178]).

The past years have shown rapid progress in identifying cargo and mechanisms of EVs in diseases; however, we believe a more detailed mechanistic and physiological understanding of signaling via EVs will be necessary to use EVs for diagnostics and therapy of cancer.

## Conflict of interest

The authors declare no conflict of interest.

## Author contributions

AS and MB wrote the manuscript. Both authors approved the final version.
